# Targeting of glioblastoma cell lines and glioma stem cells by combined PIM kinase and PI3K-p110α inhibition

**DOI:** 10.18632/oncotarget.8899

**Published:** 2016-04-21

**Authors:** Asneha Iqbal, Frank Eckerdt, Jonathan Bell, Ichiro Nakano, Francis J. Giles, Shi-Yuan Cheng, Rishi R. Lulla, Stewart Goldman, Leonidas C. Platanias

**Affiliations:** ^1^ Robert H. Lurie Comprehensive Cancer Center of Northwestern University, Chicago, IL, USA; ^2^ Division of Hematology/Oncology/Stem Cell Transplantation, Ann and Robert H. Lurie Children's Hospital of Chicago, Department of Pediatrics, Northwestern University Feinberg School of Medicine, Chicago, IL, USA; ^3^ Division of Hematology/Oncology, Northwestern University, Feinberg School of Medicine, Chicago, IL, USA; ^4^ Department of Neurological Surgery and James Comprehensive Cancer Center, The Ohio State University, Columbus, OH, USA; ^5^ Department of Medicine, Jesse Brown VA Medical Center, Chicago, IL, USA

**Keywords:** glioblastoma, PIM kinase, mTOR signaling, p110α

## Abstract

The PIM family of proteins encodes serine/threonine kinases with important roles in protein synthesis and cancer cell metabolism. In glioblastoma (GBM) cell lines, siRNA-mediated knockdown of PIM kinases or pharmacological inhibition of PIM kinases by SGI-1776 or AZD-1208 results in reduced phosphorylation of classic PIM effectors and also elements of the PI3K/mTOR pathway, suggesting interplay between PIM and mTOR signals in GBM cells. Combination of PIM kinase inhibitors with BYL-719, an inhibitor specific for the PI3K catalytic isoform p110α, results in enhanced antineoplastic effects in GBM cells. Additionally, pharmacologic inhibition of PIM kinases impairs growth of patient-derived glioma sphere cells, suggesting an important role for PIM kinases in cancer stem cell (CSC) function and survival. Such effects are further enhanced by concomitant inhibition of PIM kinase and p110α activities. Altogether these findings suggest that pharmacological PIM targeting in combination with PI3K inhibition may provide a unique therapeutic approach for the treatment of heterogeneous tumors containing populations of therapy-resistant CSCs in GBM.

## INTRODUCTION

Glioblastoma (GBM), an aggressive heterogeneous type of high-grade glioma, is generally associated with limited clinical responses to standard therapy and poor outcome. The majority of GBMs recur and the median survival of patients with GBM is about one year [1]. Novel therapeutic approaches are urgently needed to improve survival in patients with GBM. Conventional cytotoxic chemotherapies offer very limited benefit when combined with surgical resection and radiation therapy, thus molecularly targeted therapies may be particularly worthy of investigation.

Proviral insertion site in Moloney murine leukemia virus (PIM) is a group of serine/threonine kinases that have established roles in the control of signals for cellular proliferation, migration, metabolism, and survival [2]. Mice in which *PIM* kinases are knocked out are smaller in size, but still viable and fertile [3], suggesting that PIM kinases are dispensable for development. There is accumulating evidence for important roles of these kinases in survival signaling in cancer. For instance, PIM2 phosphorylates and inhibits the pro-apoptotic protein Bcl-2-associated death promoter (BAD) and also targets the eukaryotic translation initiation factor 4B (eIF4B) [4]. Accordingly, pharmacological PIM inhibition induces apoptosis and/or suppresses the proliferation of peripheral T cell lymphoma cells [5], chronic lymphocytic leukemia cells [6], and myeloid leukemia cells [7–9]. In addition to hematopoietic malignancies, PIM kinases are also overexpressed in a variety of solid tumors, including prostate and pancreatic cancer, gastric, colorectal and liver carcinomas, squamous cell carcinoma and bladder cancer [2]. PIM kinases are expressed in the brain [2], but little is known about their potential value as therapeutic targets in brain cancer.

There is some evidence suggesting that PIM and AKT kinases may recognize certain similar substrates and, in part, mediate overlapping functions [10]. Consistent with this hypothesis, AKT also targets eIF4B and BAD, which are involved in cancer cell proliferation and apoptosis, respectively [4]. AKT activation is mainly triggered by the phosphatidylinositol-4,5-biphosphate 3-kinase (PI3K). Importantly, p110α, the catalytic alpha subunit of PI3K, is consistently expressed in human GBM samples. Mutations in *PIK3CA* have been observed in up to 27% of GBM tumor samples [11–16]. Inhibition of p110α results in impaired anchorage-independent growth of GBM cells and tumor regression *in vivo* [17]. This suggests that targeting the alpha subunit of PI3K may provide a new approach for the treatment of GBM. However, it has been also recognized that pharmacological inhibition of p110α results in PI3K/AKT independent activation of mTORC1, associated with therapy resistance in breast cancer [18]. Therefore, p110α - PI3K targeting may require concomitant inhibition of survival signaling mediated by the mTOR pathway for optimal responses [18].

There has been evidence that the mTOR pathway is dysregulated/activated in GBM [19, 20], while other work has suggested that PIM1 and PIM2 are contributing to mTOR activity in hematopoietic malignant cells [21, 22]. This raises the possibility that PIM kinases may be promising targets for decreasing mTOR activity and cell proliferation in GBM. As the PI3K/AKT and PIM kinase pathways both trigger activation of the mTORC1 signaling pathway, concomitant targeting of both pathways is likely required to prevent resistance and tumor recurrence [21–23].

Tumor recurrence in GBM is largely mediated by a small population of glioma stem cells (GSCs) [24]. Importantly, the PI3K/AKT/mTOR pathway is activated in some cancer stem cells and is crucial for cancer stem cell maintenance [25]. Given the high homology of PIM and AKT substrate recognition motifs and the overlapping functions of both kinases, we sought to investigate whether concomitant inhibition of PIM kinases and the PI3K/AKT axis might be an effective strategy for inhibition of GBM cells and their respective cancer stem cells.

## RESULTS

**Figure 1:**
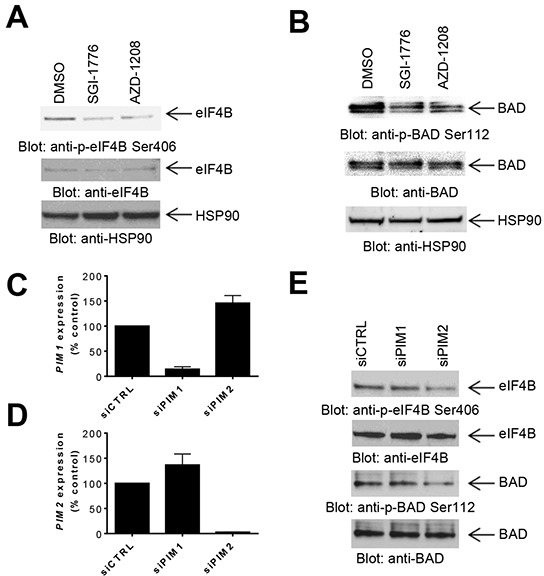
Effects of PIM kinase inhibition on phosphorylation of eIF4B and BAD in GBM cells **A–B.** LN229 cells were treated with SGI-1776 or AZD-1208 for 4 hours. Equal amounts of total cell lysates were subjected to SDS-PAGE followed by immunoblotting with the indicated antibodies to monitor phosphorylation of eIF4B (A) and BAD (B). **C–D.** siRNA mediated knockdown of PIM1 (C) or PIM2 (D), using specific siRNAs in LN229 cells. Knockdown was assessed by quantitative RT-PCR, using GAPDH for normalization. Results represent the means ± SEM of 3 independent experiments, each done in triplicates. **E.** LN229 cells were transfected with control siRNA or siRNAs directed against PIM1 or PIM2. Equal amounts of total cell lysates were subjected to immunoblotting with antibodies against the phosphorylated forms of eIF4B (pSer406) or BAD (pSer112). Equal amounts of cell lysates from the same experiment were analyzed in parallel by SDS-PAGE and immunoblotted with antibodies against eIF4B or BAD, as indicated.

**Figure 2:**
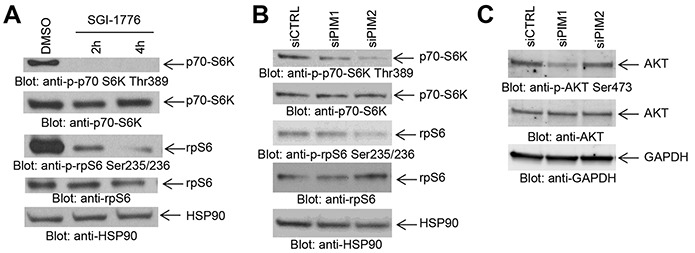
Effects of PIM kinase inhibition on mTORC1 downstream targets in GBM cells **A.** LN229 cells were treated with SGI-1776 (5 μM) for the indicated times. Equal amounts of total cell lysates were subjected to SDS-PAGE followed by immunoblotting with antibodies against the phosphorylated forms of p70-S6K (pThr389), rpS6 (pSer235/236), or against HSP90 as indicated. Equal amounts of cell lysates from the same experiment were analyzed in parallel by SDS-PAGE and immunoblotted with antibodies against p70-S6K or rpS6, as indicated. **B.** LN229 cells were transfected as indicated with control siRNA or siRNAs directed against PIM1 or PIM2. Equal amounts of total cell lysates were subjected to SDS-PAGE followed by immunoblotting with antibodies against the phosphorylated forms of p70-S6K (pThr389), rpS6 (pSer235/236), or against HSP90 as indicated. Equal amounts of cell lysates from the same experiment were analyzed in parallel by SDS-PAGE and immunoblotted with antibodies against p70-S6K or rpS6, as indicated. **C.** LN229 cells were transfected with control siRNA and siRNAs directed against PIM1 or PIM2. Equal amounts of total cell lysates were subjected to SDS-PAGE followed by immunoblotting and membranes were simultaneously incubated with antibodies directed against the phosphorylated form of AKT (pSer473), AKT or GAPDH, followed by visualization in a ChemiDoc MP Imaging System (BioRad) as described in Materials and Methods.

It has been previously demonstrated that PIM kinases phosphorylate eIF4B and BAD *in vitro* [4], but little is known regarding the substrates for PIM kinase activity in GBM cells. In initial studies we sought to determine the effects of inhibition of PIM kinases on these downstream targets. LN229 cells treated with the PIM inhibitors SGI-1776 or AZD-1208 depicted a decrease in phosphorylation of eIF4B on serine 406 (Figure 1A) and BAD on serine 112 (Figure 1B), indicating that these two known PIM effectors are also engaged in GBM cells. In further studies, we sought to dissect the contributions of distinct PIM kinase isoforms on phosphorylation of eIF4B and BAD. For this purpose, we used specific siRNAs against each isoform (Figures 1C and 1D). Knockdown of PIM2, but not PIM1, resulted in a decrease of phosphorylation of eIF4B and BAD (Figure 1E), strongly suggesting that, PIM2 is the primary isoform responsible for phosphorylation of eIF4B and BAD in LN229 GBM cells. Next, we sought to determine the effects of inhibition of PIM kinases on mTORC1 signaling. Inhibition of PIM kinases by SGI-1776 resulted in decreased phosphorylation of mTOR downstream targets p70-S6K and rpS6 (Figure 2A), suggesting that PIM kinase activity is required for phosphorylation of mTORC1 targets in GBM cells. To further identify the specific PIM kinase isoform facilitating mTORC1 signaling, LN229 cells in which PIM1 or PIM2 was knocked down were analyzed for phosphorylation of p70-S6K or rpS6. Knockdown of PIM2, but not PIM1, resulted in a substantial decrease in the phosphorylation of p70-S6K and rpS6 (Figure 2B). Notably, knockdown of PIM1 but not PIM2 decreased phosphorylation of AKT at serine 473 (Figure 2C). These results raise the possibility that PIM1 and PIM2 both enhance mTOR activity albeit through different mechanisms in GBM cells; with PIM2 primarily stimulating mTORC1 signals, while PIM1 contributes to mTORC2 activity.

**Figure 3:**
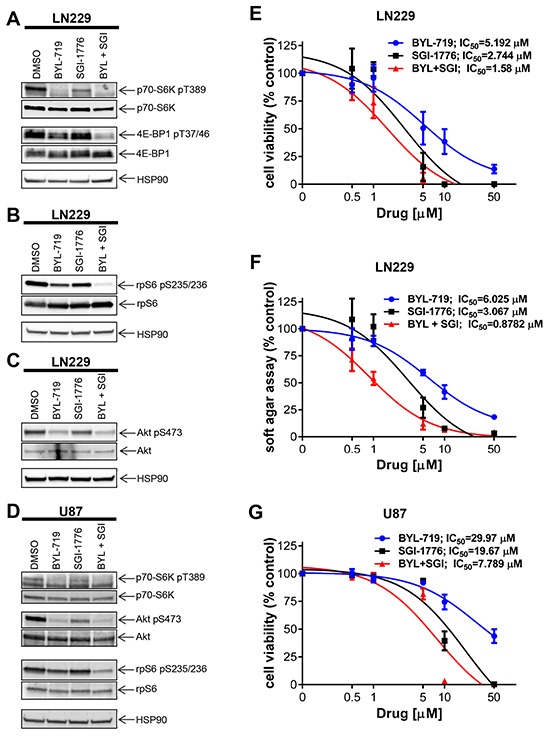
Effects of simultaneous inhibition of PIM and PI3K on cell viability and transformation of GBM cells **A.** LN229 cells were treated with SGI-1776 (5 μM) and with BYL-719 (10 μM) for 90 min. Lysates were analyzed by SDS-PAGE and immunoblotted with the indicated antibodies for HSP90 and the phosphorylated forms of p70-S6K and 4E-BP1. Subsequently, membranes were stripped and reprobed with antibodies against p70-S6K and 4E-BP1, as indicated. **B.** Same experiment as in A using antibodies for HSP90 and the phosphorylated form of rpS6 followed by stripping and reprobing with antibodies against rpS6 as indicated. **C.** LN229 cells were treated and processed as in A. Membranes were incubated simultaneously with antibodies against HSP90, the phosphorylated form of AKT on Ser473 and total AKT, followed by visualization in a ChemiDoc MP Imaging System (BioRad) as described in Materials and Methods. **D.** U87 cells were treated and processed as in A. Proteins were immunoblotted with antibodies for p70-S6K, rpS6 and their phosphorylated forms (pThr389 and pSer235/236, respectively) and HSP90 simultaneously. The membrane was stripped and reprobed with antibodies the phosphorylated form of AKT on Ser473 and total AKT simultaneously. **E.** LN229 cells were plated in 96-well plates and treated with increasing concentrations of the PIM inhibitor SGI-1776 and/or PI3K inhibitor BYL-719 for 5 days. Cell viability was assessed using WST-1 proliferation assay. Results represent the means ± SEM of 3 independent experiments, each done in triplicates. **F.** LN229 cells were plated in 96-well plates in soft agar and treated with increasing concentrations of PIM inhibitor SGI-1776 and PI3K inhibitor BYL-719 for 7 days. Colony formation was quantified using the fluorescent cell stain CyQUANT GR Dye (Cell Biolabs Inc.) in the Synergy HT Plate reader. Data are expressed as percentages of control DMSO treated samples. Results represent the means ± SEM of 3 independent experiments, each done in triplicates. **G.** Same as E using U87 cells. Cell viability was assessed using WST-1 proliferation assay. Results represent the means ± SEM of 3 independent experiments.

It has been previously suggested that resistance to PI3K inhibition in breast carcinoma may lead to therapy resistance in part by activation of the mTOR pathway [18]. There is also some evidence that PIM2 is able to promote growth and survival in the presence of rapamycin and is required to confer rapamycin resistance in malignant hematopoietic cells [26]. As our data established that PIM kinase activity modulates mTOR targets in GBM cells, we examined the possibility that PIM targeting may overcome resistance to PI3K inhibition. We examined the effects of simultaneous inhibition of PIM and PI3K on GBM cells. Combined treatment with SGI-1776 and BYL-719 resulted in more pronounced decrease in phosphorylation of p70-S6K, 4E-BP1 and rpS6 than with either drug alone (Figure 3A, 3B). BYL-719 strongly inhibited PI3K activity as indicated by reduced phosphorylation of AKT (Figure 3C). Besides LN229 cells, we also found PIM1 and PIM2 expressed in U87 cells (Supplementary Figure S1). Similar to LN229 cells, in U87 cells, BYL-719 inhibited AKT phosphorylation, while concomitant treatment with SGI-1776 led to a more pronounced decrease in phosphorylation of rpS6 and p70-S6K than either drug alone (Figure 3D). This prompted us to further examine the effects of the combination on GBM cell viability. In LN229 cells, combination of SGI-1776 with BYL-719 resulted in stronger suppression of cell viability as compared to that seen with either agent alone (Figure 3E). The half maximal inhibitory concentration (IC_50_) decreased from 2.744 μM (SGI-1776) and 5.192 μM (BYL-719) to 1.58 μM for the combination treatment (SGI-1776 and BYL-719). Subsequently, we calculated the combination index (CI) for this drug combination, which is a quantitative definition for additive effect (CI = 1), synergism (CI < 1) or antagonism (CI > 1). The CI value of 0.43609 for the combination of both drugs indicates a synergistic inhibitory effect against proliferation of LN229 cells. Similar results were obtained when the effects of the combination on anchorage-independent malignant cell growth were assessed in soft agar (Figure 3F), where the IC_50_ decreased from 3.076 μM (SGI-1776) and 6.025 μM (BYL-719) to 0.878 μM for the combination (SGI-1776 and BYL-719). The CI of 0.22302 indicates a potent synergistic effect of the drug combination on anchorage-independent growth of transformed GBM cells. Similar results were observed for cell viability in U87 cells, with a CI value of 0.76, also indicating synergistic inhibitory effects (Figure 3G).

**Figure 4:**
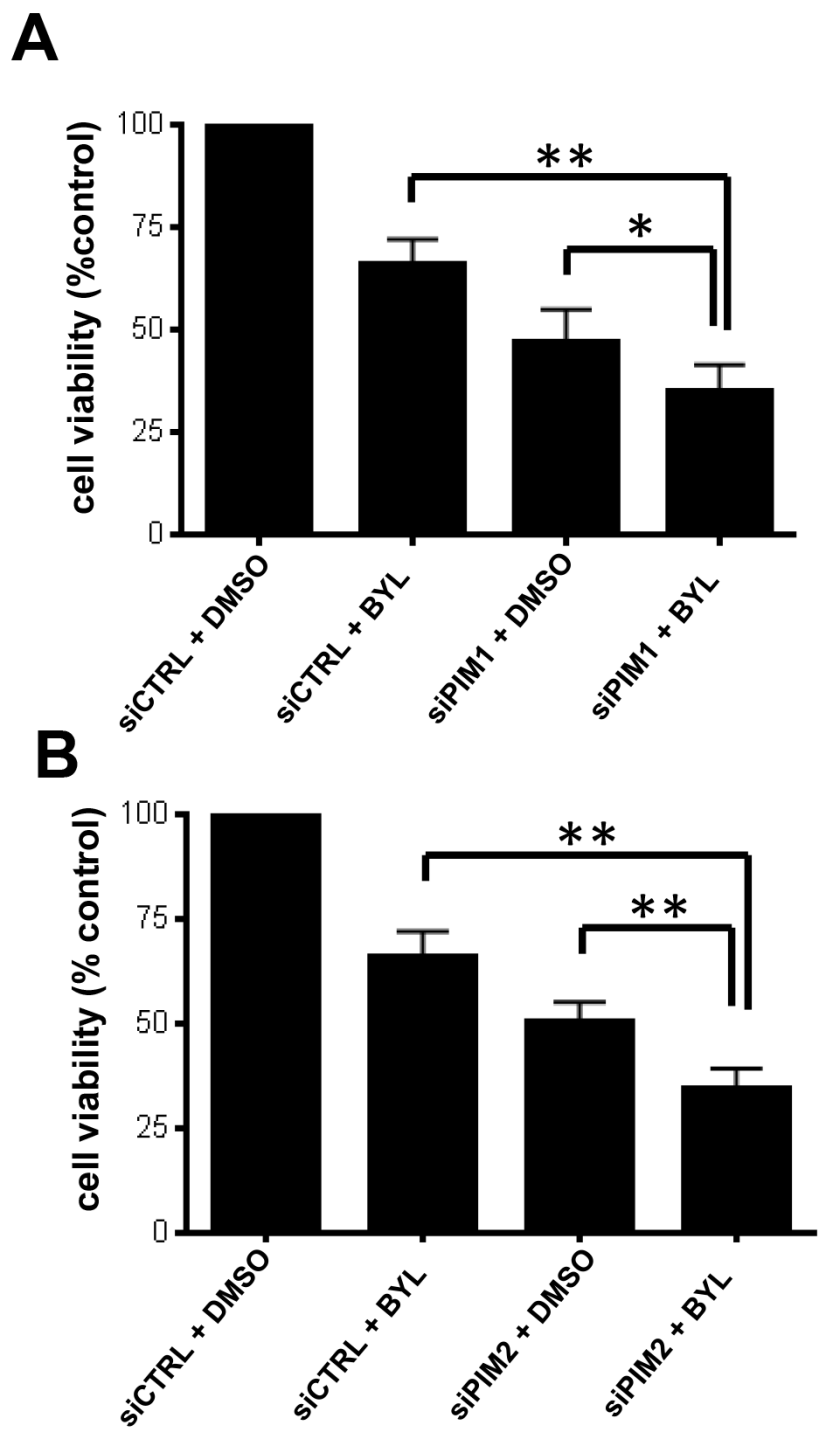
PIM knockdown sensitizes GBM cells to BYL-719 LN229 cells were transfected with control siRNA or siRNA directed against PIM1 **A.** or PIM2 **B.** After 2 days cells were seeded onto 96-well plates and treated with BYL-719. Viability was assessed 5 days later, using a WST-1 proliferation assay. *p < 0.05 **p < 0.01. Results in Figure 4A and 4B represent the means + SEM of 5 independent experiments.

Since pharmacological inhibition of PIM kinases synergistically enhanced the antineoplastic effects of BYL-719, we sought to dissect the specific roles of PIM1 and PIM2 in the process. We thus combined specific knockdown of PIM1 and PIM2 with pharmacological inhibition of PI3K using BYL-719. Knockdown of either PIM1 or PIM2, followed by treatment with BYL-719 resulted in a significant decrease in cell viability, as shown by WST-1 proliferation assay (Figure 4). When combined with BYL-719, PIM2 knockdown resulted in a more significant decrease in cell proliferation (p < 0.01), as compared to PIM1 knockdown (p<0.05).

**Figure 5:**
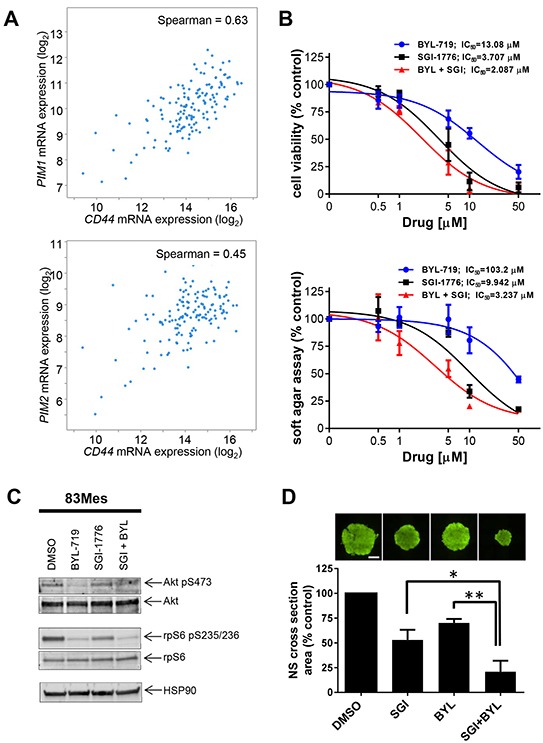
PIM kinase targeting enhances the inhibitory effects of PI3K inhibitors on patient-derived GSCs **A.** RNAseq data from n = 154 GBM patient samples (TCGA Research Network), was used to compare CD44 expression with expression of PIM1 (upper panel) or PIM2 (lower panel). Visualization of the data was performed using the cBioPortal [29, 30]. **B.** upper panel: 83Mes GBM mesenchymal stem cells were plated in 96-well plates and treated with increasing concentrations of the PIM inhibitor SGI-1776 and/or PI3K inhibitor BYL-719 for 5 days. Cell viability was assessed using WST-1 proliferation assay. Results represent the means ± SEM of 3 independent experiments. Lower panel: 83Mes GBM mesenchymal stem cells were plated in 96-well plates in soft agar and treated with increasing concentrations of PIM inhibitor SGI-1776 and PI3K inhibitor BYL-719 for 7 days. Colony formation was quantified using the fluorescent cell stain CyQUANT GR Dye (Cell Biolabs Inc.) in the Synergy HT Plate reader. Data are expressed as percentages of control DMSO treated samples. Results represent the means ± SEM of 3 independent experiments. **C.** 83Mes GBM mesenchymal stem cells grown as neurospheres were treated with SGI-1776 (5 μM) and with BYL-719 (10 μM) for 90 min. Lysates were analyzed by SDS-PAGE and immunoblotted with the indicated antibodies for HSP90, rpS6 and its phosphorylated form (pSer235/236) simultaneously. Membrane was stripped and reprobed with antibodies for AKT and its phosphorylated form (pSer473) simultaneously. **D.** 83Mes GBM mesenchymal stem cells were treated with SGI-1776 (2 μM) and/or BYL-719 (5 μM). After 7 days, neurospheres were stained with Acridine Orange at a concentration of 0.1 μg/ml for 1 hour. Subsequently, neurosphere cross-section area was determined by microscopic analysis using a Nikon Eclipse TE inverted microscope with automated stage as described in materials and methods. Representative images of neurospheres are depicted (upper panels). Scalebar = 500 μm. Means + SEM of the values from 4 independent experiments (each done in five technical replicates) are shown (lower panel). Comparisons between drug treatments were performed using paired, two-tailed t-tests, * < 0.05, ** < 0.01.

Cancer stem cells frequently overcome treatment-induced cellular damage by the currently available conventional chemotherapies, subsequently leading to lethal tumor recurrence [27]. We investigated whether PIM mRNA expression correlated with expression of a cancer stem cell marker CD44 [24]. The RNAseq data generated by TCGA Research Network (http://cancergenome.nih.gov/) using published data set classification [28] visualized using the cBioPortal [29, 30] showed a statistically significant correlation between CD44 expression and those of PIM1 (Figure 5A, upper panel) and PIM2 (Figure 5A, lower panel). Given the striking correlation of CD44 expression with PIM1 and PIM2 levels, we sought to investigate the effect of PIM inhibition on GSCs. Similar to glioblastoma cell lines, combined treatment with SGI-1776 and BYL-719 exerted synergistic antineoplastic effects on cell viability (CI = 0.90) (Figure 5B, upper panel) and colony formation (CI = 0.84) (Figure 5B, lower panel) of 83Mes GSCs. SGI-1776 and BYL-719 inhibited phosphorylation of rpS6 (Figure 5C) in GSCs. Inhibition of PI3K by BYL-719 was confirmed by reduced phosphorylation of AKT (Figure 5C). Next we investigated whether PIM inhibition can block GSC growth. 83Mes GSCs were grown as neurospheres, treated with SGI-1776 and BYL-719 individually and in combination, and subjected to the neurosphere formation assay as previously described [31]. While only minimal effect on neurosphere size by SGI-1776 or BYL-719 alone was observed, combination of SGI-1776 and BYL-719 resulted in greatly impaired neurosphere growth as indicated by a substantial decrease in neurosphere size (Figure 5D). These results strongly suggest that combining PIM and PI3K inhibitors represents a promising strategy for targeting both GBM tumor cells including their GSC population.

## DISCUSSION

In the current study we investigated the effect of PIM kinases on phosphorylation, cell proliferation, and transformation of GBM cells. Our studies using pan-PIM kinase inhibitors initially established that PIM inhibition decreases phosphorylation of known PIM targets including BAD and eIF4B in GBM cells. Notably, our studies demonstrated that knockdown of PIM2, but not PIM1, substantially decreased phosphorylation of eIF4B and BAD, suggesting that PIM2 is the main kinase responsible for phosphorylation of these targets in GBM cells.

There has been previous evidence that PIM kinases modulate mTORC1 activity [7, 9, 21, 22]. For example, PIM2 modulates mTORC1 activity through phosphorylation of TSC2 in other cancers [21], while PIM1 phosphorylates PRAS40 thereby relieving its inhibitory effects on mTORC1 with a subsequent increase in mTORC1 activity [22]. As there is extensive evidence that the mTOR pathway is constitutively active in GBM [19, 20, 32, 33], we sought to determine the effect of inhibition of the PIM kinase pathway on downstream mTORC1 effectors such as p70-S6K and rpS6. We found that PIM inhibition results in decreased phosphorylation of mTORC1 effectors p70-S6K, 4E-BP1 and rpS6, suggesting that PIM kinases enhance mTORC1 activity in GBM cells. Such effects appear to selectively reflect PIM2 activity, as unlike the results of PIM1 knockdown, PIM2 knockdown decreased phosphorylation of these targets. PIM1 may be involved in the stimulation of mTORC2 complexes, as suggested by the inhibitory effects of PIM1 knockdown on phosphorylation of AKT on serine 473, a phosphorylation site that is under the control of mTORC2 [23, 34, 35]. Thus, the PIM pathway seems to be required for optimal engagement of mTOR targets in GBM, in line with observations from other cancers [21, 22]. The mTOR pathway drives cancer cell metabolism and the efficiency of mTOR inhibition in cancer can be enhanced by subsequent PI3K inhibition, which led to the development of dual ATP-competitive PI3K and mTOR inhibitors. Recently, specific inhibition of the PI3K catalytic isoform p110α was found to impair GBM cell proliferation and anchorage-independent cell growth [17]. This suggests that selective inhibition of p110α might represent a promising strategy for GBM and in particular, for targeted combinatorial approaches. In efforts to determine whether PIM kinase inhibition can enhance the effects of the PI3K p110α inhibitor BYL-719, we found potent enhancing effects on cell viability and anchorage-independent growth of GBM cells. Importantly, combined inhibition of p110α and PIM synergistically suppressed both GBM cell viability and transformation, providing a rationale for this novel combination in the treatment of GBM.

The main cause for mortality in GBM is recurrence of the tumor after therapeutic failure, which is attributed, at least in part, to a small subpopulation of therapy-resistant GSCs that are able to self-renew and repopulate the recurrent tumor [27]. Little is known about the role of PIM kinases in CSC function in solid tumors. Using The Cancer Genome Atlas (http://cancergenome.nih.gov/), we were able to demonstrate that expression of both PIM1 and PIM2 mRNA in GBM correlates with that of a stem cell marker CD44, supporting a possible role for PIM kinases in cancer stem cell function. Consistent with this, pharmacologic inhibition of PIM kinases substantially reduced growth of patient-derived GBM neurospheres in culture and this inhibitory effect was significantly enhanced when the PI3K isoform p110α was inhibited concomitantly. These results strongly suggest that both p110α and PIM kinases play synergistic roles in the biology of GSCs and provide the basis for a unique approach to eliminate malignant GSCs involving simultaneous targeting of both the PIM kinase and PI3K pathways.

## MATERIALS AND METHODS

### Cell lines, reagents, antibodies, and inhibitors

LN229 and U87 GBM cells were maintained in DMEM supplemented with 10% fetal bovine serum and antibiotics at 37°C in 5% CO_2_. Maintenance of 83Mes GSCs was done as described elsewhere [24]. The antibodies against p-p70-S6K (Thr389), p-AKT (Ser473), p-eIF4B (Ser406), p-rpS6 (Ser235/236), p-4E-BP1 (Thr37/46), p-BAD (BAD Ser112), eIF4B, p70-S6K, AKT, BAD, rpS6, 4E-BP1 and mTOR were obtained from Cell Signaling Technology (Danvers, MA). Monoclonal mouse anti-p70-S6K and rabbit anti-HSP90 antibodies were from Santa Cruz Biotechnology (Santa Cruz, CA) and GAPDH antibody was from Millipore (Billerica, MA). For gene silencing of PIM1 and PIM2 by siRNA, cells were transfected with double stranded control non-targeting or PIM1 or PIM2 targeting RNA oligonucleotides (Santa Cruz Biotechnology, Santa Cruz, CA.). For transfection, Lipofectamine RNAiMAX Reagent (Invitrogen, Carlsbad, CA) was used, according to the manufacturer's instructions. The SGI-1776 and AZD-1208 pan PIM kinase inhibitors were obtained from Selleckchem (Houston, TX) and AstraZeneca (Wilmington, DE), respectively. BYL-719 is a PI3 kinase inhibitor, exhibiting specificity towards the p110α subunit of PI3 kinase, and was obtained from Chemietek (Indianapolis, IN). SGI-1776, AZD-1208 and BYL-719 were used at final concentrations of 10 μM, unless otherwise indicated.

### Cell lysis and immunoblotting

After the indicated treatments, cells were lysed in phosphorylation lysis buffer containing protease inhibitors and phosphatase inhibitors, and prepared for immunoblotting as in our previous studies [36, 37]. In some cases membranes were simultaneously incubated with antibodies against the phosphorylated protein (produced in rabbit) and antibody against the total protein (produced in mouse) followed by simultaneous detection with secondary anti-rabbit-HRP and anti-mouse-Alexa Fluor 488 antibody, visualized in a ChemiDoc MP Imaging System (BioRad).

### Cell viability/proliferation assays

Experiments using the 3-(4,5-dimethyl-2-thiazolyl)- 2,5-diphenyltetrazolium bromide (MTT) methodology were carried out using the WST-1 assay kit (Roche, Mannheim, Germany), according to the manufacturer's instructions. In brief, LN229, U87 or 83Mes cells were seeded at 2000 cells per well in a 96-well plate and incubated with the indicated inhibitors. For siRNA experiments, cells were transfected with the indicated siRNAs 2 days prior to seeding into 96-well plates, followed by treatment with the indicated agents. After 5 days, 10% (v/v) WST-1 reagent was added to each well and absorbance at 450 nm was analyzed (using absorbance at 600 nm as a reference wavelength), using an Epoch Plate reader and Gen5 software from BioTek Instruments Inc.

### Soft agar assays

For investigation of anchorage-independent cell growth, soft-agar assays were performed using the CytoSelect 96-Well Cell Transformation Assay Kit (Cell Biolabs, Inc.) according to the manufacturer's instructions. In brief, LN229 or 83Mes cells were seeded in soft-agar in a 96-well plate and incubated at 37°C in 5% CO_2_ with the indicated inhibitors. After 7 days, agar was solubilized and cells were lysed according to the manufacturer's instructions. Colony formation was quantified using the fluorescent cell stain CyQUANT GR Dye (Cell Biolabs Inc.) in the Synergy HT Plate reader using Gen5 software from BioTek Instruments Inc.

### Quantitative real time PCR

mRNA was reverse-transcribed into cDNA using the Omniscript RT kit (Qiagen) and oligo(dT) primers (Life Technologies) as in our previous studies [38]. Quantitative PCR using commercially available Taqman primers (Applied Bio-systems) was used to determine *PIM1* and *PIM2* mRNA expression, using *GAPDH* for normalization.

### Statistical analysis

Statistical analysis was performed using Prism Graphpad 6 for PC, including calculation of IC_50_ values by a sigmoidal dose-response curve fit. CI values were calculated for the IC_50_ values using Compusyn to determine whether drug interactions were additive (CI = 1), synergistic (CI < 1) or antagonistic (CI > 1) [39]. Correlation was assessed by nonparametric Spearman test using the cBioPortal website [29, 30].

### Neurosphere assays

Neurosphere assays were performed as described previously [31] with the exception that we used 83Mes (mesenchymal) patient-derived glioma stem cells [24]. In brief, 83Mes cells were plated into a round bottom 96-well plate at 500 cells per well. Subsequently, cells were treated with indicated inhibitors. After seven days, neurospheres were stained with Acridine Orange (0.1 μg/ml) and subjected to microscopic analysis to determine neurosphere cross-section area as described previously [31].

## SUPPLEMENTARY FIGURE



## Abbreviation

BAD: Bcl-2-associated death promoter
CSC: cancer stem cell
eIF4B: eukaryotic translation initiation factor 4B
GBM: glioblastoma multiforme
GSC: glioma stem cell
mTOR: mammalian target of rapamycin
PI3K: phosphatidylinositol-4,5-bisphosphate-3-kinase
PIM: Proviral insertion site in Moloney murine leukemia virus
rpS6: ribosomal protein S6
p70-S6K: ribosomal protein S6 kinase
