# Supplementary material for: Targeting of glioblastoma cell lines and glioma stem cells by combined PIM kinase and PI3K-p110α inhibition
Source: Oncotarget. 2016 Apr 21;7(22):33192–201. doi: 10.18632/oncotarget.8899 (PMC5078085; doi:10.18632/oncotarget.8899)
Supplement: Supplementary file 1 [file oncotarget-07-33192-s001.pdf]

SUPPLEMENTARY FIGURE

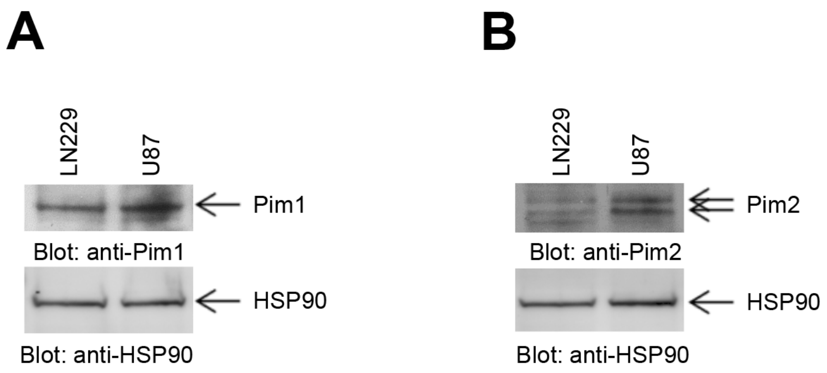

Supplementary Figure S1: PIM protein expression in GBM cell lines. A. LN229 and U87 GBM cells were subjected to Western Blot analysis using antibodies for PIM1 and HSP90 (A) or PIM2 and HSP90 **B**.
